# Two-Person Technique of Peroral Endoscopic Myotomy for Achalasia with an Advanced Endoscopist and a Thoracic Surgeon: Initial Experience

**DOI:** 10.1155/2016/2656101

**Published:** 2016-08-17

**Authors:** Madhusudhan R. Sanaka, Ramprasad Jegadeesan, Prashanthi N. Thota, Udayakumar Navaneethan, Rocio Lopez, Sudish C. Murthy, Siva Raja

**Affiliations:** ^1^Department of Gastroenterology, Cleveland Clinic, Cleveland, OH 44195, USA; ^2^Department of Thoracic Surgery, Cleveland Clinic, Cleveland, OH 44195, USA

## Abstract

*Background and Aims*. We initiated peroral endoscopic myotomy (POEM) utilizing a two-person technique with combination of an advanced endoscopist and a thoracic surgeon with complementary skills. Our aim was to determine the feasibility and outcomes in initial 20 patients*. Methods*. In this observational study, main outcomes measured were therapeutic success in relieving symptoms (Eckardt score < 3), decrease in lower esophageal sphincter (LES) pressures, improvement in emptying on timed barium esophagogram (TBE), and complications.* Results*. POEM was successful in all 20 patients with a mean operative time of 140.1 + 32.9 minutes. Eckardt symptom scores decreased significantly at two-month follow-up (6.4 + 2.9 versus 0.25 + 0.45, *p* < 0.001). Both basal and residual LES pressures decreased significantly (28.2 + 14.1 mmHg versus 12.8 + 6.3 and 22.4 + 11.3 versus 6.3 + 3.4 mmHg, *p* = 0.025 and <0.001, resp.). Barium column height at 5 minutes on TBE reduced from 6.8 + 4.9 cm to 2.3 + 2.9 cm (*p* = 0.05). Two patients (10%) had mucosal perforations and one had delayed bleeding (5%).* Conclusions.* Two-person technique of POEM with combination of an advanced endoscopist and a thoracic surgeon is highly successful with low risk of complications.

## 1. Introduction

Achalasia is a primary esophageal motility disorder characterized by esophageal aperistalsis and incomplete lower esophageal sphincter (LES) relaxation during swallowing. Classical symptoms include dysphagia, regurgitation, chest pain, and weight loss. Goal of current treatment modalities of achalasia is to decrease the lower esophageal sphincter pressure, thereby relieving the symptoms. Standard treatments include pneumatic dilation, Heller myotomy, and Botulinum toxin injection into LES [1]. Pneumatic dilation and Heller myotomy have comparable midterm efficacy, and Botulinum toxin injection provides only temporary relief [1, 2]. Peroral endoscopic myotomy (POEM) is* the new kid on the block* and is evolving as a very promising treatment modality for achalasia.

POEM is a novel, incisionless treatment modality that combines the efficacy of a surgical myotomy with the convenience of an endoscopic procedure. The concept of POEM is derived from Natural Orifice Transluminal Endoscopic Surgery (NOTES) and advances in endoscopic submucosal dissection (ESD) techniques. Feasibility of endoscopic esophageal myotomy in an animal model was initially described by Pasricha et al. and it was refined and translated into clinical practice by Inoue et al. [3, 4]. Performing a POEM requires technical skills that encompass both endoscopy and surgery. It requires advanced endoscopic skills similar to ESD, surgical skills including thorough knowledge of surgical anatomy of esophageal and mediastinal structures, and the ability to manage adverse events [5]. Hence, we started POEM program at our institution in 2014, utilizing a two-person technique with combination of an advanced gastrointestinal endoscopist and a thoracic surgeon with complementary skill sets. Our multidisciplinary approach encompasses standardized protocol, including careful patient selection after thorough diagnostic evaluation, standardized POEM technique, immediate post-POEM care, and subsequent follow-up care.

POEM is rapidly gaining popularity and is being performed at increasing number of tertiary centers around the world [6]. Currently, most of the reported outcomes of POEM for achalasia are from those performed by either foregut surgeons, thoracic surgeons with endoscopic training, or gastroenterologists with training in POEM and/or ESD [6]. There are reports of two operators performing the procedure together; however, both of the operators are usually from the same specialty [7, 8]. Aim of this study was to report the short-term outcomes from our initial 20 consecutive achalasia patients who underwent POEM by our two-person technique with combination of an advanced gastrointestinal endoscopist and a thoracic surgeon.

## 2. Methods

Data on initial 20 consecutive patients who underwent POEM for achalasia by our two-person technique at our institution from April to October 2014 were collected. Patients were identified from our Institutional Review Board approved prospective POEM registry. Patient demographics, clinical history, preoperative and procedural data, complications, and follow-up data were collected from our secure REDCAP database.

### 2.1. Our Standard POEM Protocol

#### 2.1.1. Procedure Training and Credentialing

POEM operators at our institution (MS, SR, and SM) received adequate training and practice of POEM in ex vivo porcine model, attended live POEM courses, and observed POEM procedures performed by experienced operators before starting human cases. MS has also visited Japan for 2 weeks and obtained training in ESD and POEM by observership before starting POEM at our institution. Each of our POEM operators performed at least 15 POEM procedures in ex vivo porcine esophageal model before performing human cases. Credentialing process at out institution involved at least two proctored cases initially by an experienced POEM operator before performing the POEM independently. Hence, our first two cases were proctored by an external experienced operator.

#### 2.1.2. Initial Evaluation and Patient Selection

All patients were evaluated by a multidisciplinary team comprising gastroenterologists with expertise in esophageal diseases, thoracic surgeons, and radiologists. Patients had a standard diagnostic work-up prior to POEM including an upper endoscopy, TBE, HREM, and Eckardt symptom scoring and staging of achalasia. Achalasia was staged according to the Eckardt score as stage 0 (score 0-1), stage 1 (score 2-3), stage 2 (score 4–6), and stage 3 (score >6) [9]. Initially the following subsets of patients were considered for POEM: (a) patients in whom laparoscopic Heller myotomy is technically difficult or less desirable such as obese patients, patients with multiple upper abdominal surgical scars, that is, hostile abdomen, and those with prior failed Heller myotomy and (b) patients over 60 years of age (not younger patients since long-term cumulative effects of GERD after POEM are not yet known). Selected patients underwent POEM based on the consensus reached at the multidisciplinary meeting and after discussion with the patient about the alternatives such as Heller myotomy and pneumatic dilation.

#### 2.1.3. Pre-POEM Preparation

All patients were instructed to be on clear liquid diet for 3 days prior to procedure. Nystatin oral suspension (100,000 units/mL) was prescribed for 3 days prior to procedure at a dose of 2 mL every 6 hours to swish and swallow orally. They were given half a gallon of Golytely (PEG-electrolytes) oral solution for bowel cleansing on the night before the procedure similar to those undergoing Heller myotomy at our institution. Patients fasted past midnight on the day prior to the procedure. All patients received antibiotic prophylaxis with 1500 mg of intravenous cefuroxime and 200 mg of intravenous fluconazole 30 minutes prior to POEM.

#### 2.1.4. POEM Procedure

All procedures were performed in an operative room under general anesthesia by standard technique similar to that described by Inoue et al. [4]. Carbon dioxide insufflation was used instead of room air with a carbon dioxide insufflator (UCR, Olympus Co., Tokyo, Japan). All procedures were performed with a high-definition endoscope (GIF H-190, Olympus Co., Tokyo, Japan). After insertion of an endoscope, esophageal mucosa on the anterior wall (posterior wall in patients with prior Hellers myotomy), 10–14 cm proximal to gastroesophageal junction (GEJ), was lifted with submucosal injection of 10 mL of indigo carmine stained normal saline. A 2 cm longitudinal mucosotomy incision was performed with a triangle-tip knife (TT-knife, Olympus Co., Tokyo, Japan) with the Endocut current, setting 2-1-3 (VIO300D, ERBE, Elektromedizin GmbH, Tubingen, Germany). Then, submucosal tunnel was created using spray coagulation (effect 2, 50 watts) and extended 2–4 cm onto the gastric side. Selective myotomy of inner circular muscle fibers (at times resulting in complete myotomy including longitudinal fibers) was performed using spray coagulation starting 5–8 cm proximal to GEJ and extending 2–4 cm onto the gastric wall. Any large bleeding vessels were coagulated using Coagrasper forceps (FD 410-LR, Olympus Co., Tokyo, Japan). Before the end of the procedure, 40 mg of gentamycin mixed with 20 mL of normal saline was sprayed into the submucosal tunnel. Finally, the mucosotomy was closed with endoscopic clips (Instinct Clips, Cook Endoscopy, Winston-Salem, NC, USA). During the procedure, if there was significant capnoperitoneum as evidenced by increase either in plateau pressure on mechanical ventilator or on subjective palpation of abdomen, an 18-gauge angiocath needle (or Veress needle) was placed in the right subcostal area to decompress the peritoneum.

During our lab experience/practice we found that having a second operator holding and stabilizing the endoscope shaft while the primary operator was controlling the dials and devices was very helpful. Hence, we adopted the two-person POEM technique. In our two-person technique, both the advanced endoscopist and the thoracic surgeon performed the procedure together with each one performing certain portions of the procedure alternatively. The primary operator worked with the endoscope dials and catheters/devices. The second operator held and stabilized the endoscope shaft while applying constant tension at the submucosa-muscularis propria interface for faster and safer dissection. Mucosotomy, submucosal tunneling, and endoscopic clipping were mostly performed by the advanced endoscopist. Thoracic surgeon mostly performed myotomy and ensured adequacy of myotomy in terms of both completeness and appropriate length onto the gastric side. However, these steps were interchangeable and arbitrary between the two operators in different procedures. Operative time was measured from initial insertion of endoscope until the end of the procedure.

#### 2.1.5. Perioperative Care

All patients were kept nil per os (NPO) and were observed overnight in the hospital on intravenous fluids, intravenous proton pump inhibitor, and an intravenous antibiotic, cefuroxime. A gastrografin swallow study was performed next day and patients were started on clear liquid diet if the swallow study did not show a leak and were discharged home. The diet was gradually advanced over next several days. A proton pump inhibitor was prescribed to all patients for at least 2 months.

#### 2.1.6. Follow-Up Care

A follow-up was arranged in 1-2 weeks to evaluate for any adverse events. Then another follow-up visit was arranged at about 2 months after procedure. At that time, evaluations included Eckardt symptom scoring, timed barium esophagogram (TBE), 24-hour esophageal pH study of medications, and a high-resolution esophageal manometry (HREM) study. Patients with either gastroesophageal reflux disease (GERD) symptoms or abnormal 24-hour pH study were continued on PPI therapy indefinitely. A 24-hour pH study was considered abnormal if esophageal acid exposure was greater than 4.5% of the total time. An Eckardt symptom score of ≤3 was considered as therapeutic success or remission.

#### 2.1.7. Statistical Analysis

Data are presented as mean ± standard deviation, median [P25, P75], or *N* (%). A subgroup analysis was performed in subjects who had 2-month postprocedure follow-up. Differences between pre- and postprocedure were assessed with one-sample *t*-test or the nonparametric Wilcoxon signed rank test for continuous factors and test of symmetry for categorical factors. A *p* ≤ 0.05 was considered statistically significant. All analyses were performed using SAS (version 9.4, The SAS Institute, Cary, NC).

## 3. Results

The mean age of this patient cohort was 56.7 years (±18.1). The male to female ratio was 14 to 6. Most of the patients were Caucasian (17/20, 85%) and the mean BMI was 28.6 (±5.9). ASA class was II in 7 patients, III in 11 patients, and IV in 2 patients, respectively. Preprocedural characteristics including achalasia types, findings on HREM, TBE, Eckardt symptom scores and stage, and details of prior treatments received for achalasia are shown in Table 1. Only 30% of the patients were treatment naïve and the rest received either one or multiple standard treatments of achalasia prior to POEM. More than 50% of patients had advanced stage 3 achalasia. One patient (5%) had sigmoid esophagus. POEM procedural data are summarized in Table 2. POEM was successfully performed in all 20 patients (100%). Mean operative time was 140.1 ± 32.9 minutes. Five patients (25%) needed Veress needle placement to decompress capnoperitoneum. Six patients (30%) with history of prior Heller myotomy had mucosotomy and submucosal tunnel created on the posterior esophageal wall while the rest of the 14 patients (70%) had standard anterior wall procedure.

Two patients with prior Heller myotomy had inadvertent mucosal perforations near the GEJ. One of them could be closed with one endoscopic clip and the other one was larger than 1 cm and was not amenable to clipping. Both of these patients were kept NPO for two days after POEM (instead of our standard 1 day) and then had a barium swallow study which was negative for a leak. They were started on clear liquids and were discharged home. Median length of hospital stay of our patients after POEM was 1 day (1.00 and 2.5) and return to activities of daily living (ADL) was 5 days (5.0 and 7.0). One patient (one of the two patients with a large mucosal perforation at GEJ during POEM) required hospitalization on 19th postoperative day, due to melena with a 4 gram drop in hemoglobin level. He was noncompliant with the prescribed proton pump inhibitor. An upper endoscopy performed showed a 1 cm clean based ulcer at the GEJ, most likely source of bleeding. He did not require any blood transfusion and was discharged home within 2 days.

Comparative data between pre- and post-POEM at two-month follow-up are summarized in Table 3. All 12 patients with a two-month follow-up had remission of symptoms with reduction in Eckardt score to 0.25 ± 0.45 post-POEM versus 6.4 ± 2.9 pre-POEM. There was significant improvement in HREM parameters including resting LES pressure (12.8 ± 6.3 versus 28.2 ± 14.1 mmHg) and integrated residual LES pressure (IRP) (6.3 ± 3.4 versus 22.4 ± 11.3 mmHg). Most of the parameters at both one minute and 5 minutes improved significantly in TBE as well. GERD symptoms were reported by 4 out of 11 patients (36.4%). Twenty-four-hour esophageal pH study was abnormal with increased acid exposure in 5 out of 10 patients (50%).

## 4. Discussion

Our study showed that performance of POEM with two-person technique by an advanced endoscopist and a thoracic surgeon was highly successful with excellent short-term outcomes in our initial 20 patients. This was despite our patients being more complex compared to other reported early series, with majority of them with advanced achalasia and failure of other standard treatment modalities.

This study's findings are similar to previous reported studies in terms of short-term outcomes including treatment success and low risk of complications [8, 10–12]. Most of our patients had significant improvement in symptoms reflected by significant reductions in Eckardt scores. There was also physiological improvement in esophageal function as evidenced by decreased LES pressures on follow-up HREM and improved esophageal emptying on TBE. GERD symptoms were reported by 36% of patients and this is similar to other published studies from the western world [8, 11, 12]. Two patients (10%) with prior Heller myotomy had mucosal perforations near GEJ on the gastric side during POEM. Inadvertent mucosal perforations are known to occur and our incidence is similar to other reported studies [8]. One patient had delayed bleeding, most likely from a GEJ ulcer. Noncompliance with proton pump inhibitor might have contributed to this complication. GEJ ulcers were reported in about 20% of patients after POEM probably related to thermal injury during POEM or due to ischemia [8].

Our study patients were more complex compared to other reported early series. Only 30% of our patients were treatment naïve and the rest of the patients have failed other treatment modalities of achalasia. Some of them have received multiple treatments prior to POEM. Since POEM is a complex procedure requiring high technical skills and a long learning curve, many experts recommend beginners to perform treatment naïve cases for up to 20 procedures before taking on complex cases [13, 14]. However, this was not feasible as most of the achalasia patients referred to us have failed prior treatments. Despite this, we were able to perform POEM successfully in these complex patients using our meticulous combined approach. From our study experience, we recommend that complex POEM cases should be performed only in tertiary care centers with availability of expertise to handle untoward complications.

Although POEM can be performed by a single operator, we believe our two-person technique has several inherent advantages. The main advantage is the constant tension applied to the submucosa-muscularis propria interface by the second operator in the two-person technique, which is not easily accomplished by the single operator technique. This facilitates safer and faster dissection with more control. Additionally, in complex POEM procedures such as those performed after numerous endoscopic interventions or after failed Heller myotomy, the added experience of two physicians over that of the single operator has value in appropriately identifying planes when they are not very clear. This is especially important in the early experience of operators in this relatively new technique. Having a second operator might also help in reducing operator fatigue and ensuring that each step is done thoroughly. If two-person technique is not feasible and POEM is to be performed by a gastroenterologist, we suggest having a thoracic or foregut surgery backup available as needed.

Some of our study limitations include small sample size and short follow-up. We do not know if these outcomes persist in the long run. However, it should be noted that POEM was successfully performed in advanced achalasia patients that have failed other conventional treatment modalities including Heller myotomy. Treatment options for such patients are limited and include morbid redo Heller myotomy or esophagectomy. POEM is an excellent minimally invasive rescue option for them. Another limitation of our two-person technique is the need to coordinate the schedules of the two operators to perform the procedure. At our institution, both operators bill the procedure as cosurgeons and share the revenue. We plan to continue this approach at our institution due to the complimentary skill sets of the operators and excellent outcomes. However, this approach might be very useful for initiating a POEM program until the operators get through the initial learning curve of 20 cases or so. This study is from a tertiary care referral center in which two experienced operators performed all the procedures, hence these findings may not be generalizable to general community practices. However, since achalasia is a rare condition, we do not foresee community practitioners performing this procedure.

In conclusion, our findings suggest that two-person technique of POEM by an advanced gastrointestinal endoscopist and a thoracic surgeon is highly successful with excellent short-term outcomes. Although POEM is a low risk procedure, serious complications such as severe bleeding, mediastinitis, and potential damage to surrounding vital structures can occur [15]. POEM is still an evolving treatment for achalasia with unknown long-term side effects, especially from GERD such as possible Barrett's esophagus and esophageal adenocarcinoma. These possibilities should be discussed with patients before considering POEM.

## Figures and Tables

**Table 1:** Pre-POEM characteristics.

Factor	*n*	Summary
Achalasia types	20	
(i) Type 1		9 (45)
(ii) Type 2		10 (50)
(iii) Type 3		1 (10)
Basal LES pressure (mmHg)	14	35.9 ± 20.4
LES residual pressure (mmHg)	18	23.6 ± 10.4
Barium esophagogram findings		
Column height at 1 min (cm)	18	7.5 ± 5.4
Column width at 1 min (cm)	18	3.6 ± 2.4
Volume remaining at 1 min (cc)	18	59.1 [25.1, 127.2]
Column height at 5 min (cm)	18	6.3 ± 5.5
Column width at 5 min (cm)	18	2.8 ± 1.7
Volume remaining at 5 min (cc)	18	48.9 [21.3, 88.3]
Maximal esophageal width (cm)	16	3.8 ± 2.3
Duration of achalasia symptoms (years)	20	3.0 [1.8, 6.0]
Eckardt dysphagia score	19	2.3 ± 0.82
Eckardt chest pain score	19	1.00 ± 1.00
Eckardt regurgitation score	19	1.8 ± 0.76
Eckardt weight loss score	19	1.5 ± 1.2
Total Eckardt score	19	6.7 ± 2.5
Eckardt staging	19	
(i) Stage 1		3 (15.8)
(ii) Stage 2		6 (31.6)
(iii) Stage 3		10 (52.6)
Prior treatments		
None	20	6 (30.0)
Botulinum toxin	20	4 (20.0)
Pneumatic dilation	20	8 (40.0)
Heller myotomy	20	6 (30.0)
Number of prior treatments	19	
(i) 0		6 (31.6)
(ii) 1		8 (42.1)
(iii) 3		3 (15.8)
(iv) 4		1 (5.3)
(v) 8		1 (5.3)

Values presented as mean ± SD, median [P25, P75], or *N* (column%).

**Table 2:** POEM procedure details.

Factor	*n*	Summary
Operative time (minutes)	19	140.1 ± 32.9
Submucosal tunnel length (cm)	20	15.7 ± 2.3
Myotomy length, on esophageal side (cm)	20	5.6 ± 2.2
Myotomy length, on gastric side (cm)	20	4.3 ± 1.03
Total myotomy length (cm)	20	9.8 ± 2.1
Number of endoscopic clips used to close mucosotomy	20	7.2 ± 1.8
Mucosotomy site distance proximal to GEJ (cm)	20	10.4 ± 3.2

Values presented as mean ± SD or *N* (column%).

**Table 3:** Pre- versus post-POEM characteristics on 2-month follow-up.

Factor	*n*	Pre-POEM	Post-POEM	Pre-post difference	*p* value
TBE findings					
Column height at 1 min (cm)	11	7.5 ± 4.5	4.7 ± 4.3	3.1 ± 5.7	0.12^a^
Column width at 1 min (cm)	11	3.2 ± 1.3	1.7 ± 1.6	1.5 ± 1.2	0.005^a^
Volume remaining at 1 min (cc)	11	52.3 [25.1, 106.0]	17.7 [0.00, 49.2]	40.6 [0.81, 79.1]	0.027^b^
Column height at 5 min (cm)	11	6.8 ± 4.9	2.3 ± 2.9	4.3 ± 6.0	0.05^a^
Column width at 5 min (cm)	11	2.8 ± 1.4	0.99 ± 1.2	1.7 ± 1.2	0.002^a^
Volume remaining at 5 min (cc)	11	47.1 [21.3, 76.9]	0.00 [0.00, 26.7]	24.5 [19.6, 52.1]	0.004^b^
Maximal esophageal width (cm)	9	3.1 ± 1.2	2.1 ± 1.6	0.77 ± 0.74	0.033^a^
HREM findings					
Resting LES pressure (mmHg)	7	28.2 ± 14.1	12.8 ± 6.3	22.5 ± 14.4	0.025^a^
Residual LES pressure (mmHg)	7	22.4 ± 11.3	6.3 ± 3.4	20.3 ± 9.2	0.001^a^
Eckardt symptom scores					
Eckardt dysphagia score	12	2.2 ± 0.94	0.08 ± 0.29	2.1 ± 1.00	<0.001^a^
Eckardt chest pain score	12	1.08 ± 1.2	0.17 ± 0.39	0.92 ± 1.2	0.026^a^
Eckardt regurgitation score	12	2.0 ± 0.85	0.00 ± 0.00	2.0 ± 0.85	<0.001^a^
Eckardt weight loss score	12	1.2 ± 1.2	0.00 ± 0.00	1.2 ± 1.2	0.006^a^
Eckardt total score	12	6.4 ± 2.9	0.25 ± 0.45	6.2 ± 2.9	<0.001^a^
GERD symptoms	11	0	4 (36.4)		
24-hour pH study	10				
Normal			5 (50)		
Abnormal			5 (50)		

Values presented as mean ± SD, median [P25, P75], or *N* (column%). *p* values: a: one-sample test for pre-post difference; b: Wilcoxon signed rank test for pre-post difference.

## Abbreviations

HREM: High-resolution esophageal manometry
TBE: Timed barium esophagogram
POEM: Peroral endoscopic myotomy
ASA: American Society of Anesthesiologists
EGD: Esophagogastroduodenoscopy
LES: Lower esophageal sphincter
ADL: Activities of daily living
LOS: Length of stay
NPO: Nil per os
GERD: Gastroesophageal reflux disease.
